# Intravascular Hemolysis in Aluminum Phosphide Poisoning

**DOI:** 10.5005/jp-journals-10071-23128

**Published:** 2019-02

**Authors:** Sayan Malakar, Bhagwan Dass Negi, Katyayani Dutt, Sujeet Raina

**Affiliations:** 1-4 Department of Medicine, Dr RPGMC, Tanda, Kangra, HP, India

**Keywords:** Aluminum phosphide, Hemolysis, Intravascular, Poisoning

## Abstract

Hemolysis associated with aluminum phosphide poisoning is very rare. Intravascular hemolysis in presence of glucose-6-phosphate dehydrogenase (G6PD) deficiency has been reported rarely in literature. We are reporting a case of young male patient with history of aluminum phosphide poisoning and complicated with intravascular hemolysis without G6PD deficiency. It has been reported only once in the literature.

**How to cite this article:**

Malakar S, Dass B *et al*. Intravascular Hemolysis in Aluminum Phosphide Poisoning. Indian J of Crit Care Med 2019;23(2):106-107.

## INTRODUCTION

Aluminum phosphide(ALP) is cheap and easily available fumigant pesticide commonly used in rural india. After ingestion of the substance the mortality is very high as there is no known antidote^1^. Management includes mainly supportive with intravenous fluid, ionotropes, magnesium sulfate and high dose steroids. ALP reacts with water and releases phosphine gas which inhibits mitochondrial oxidative phosphorylation and protein synthesis leading to diffuse cellular dysfunction and organ failure. It also participates in free radical induced toxicity^2^. Cardiorespiratory dysfunction is the principal reason for mortality. Rarely, intravascular hemolysis has been reported in patients with aluminum phosphide poisoning and associated with G6PD deficiency^3,4^. Here we are presenting an unusual complication of intravascular hemolysis in aluminum phosphide poisoning without G6PD deficiency. This is an extremly rare presenation and reported only once in literature^5^. The patient was managed conservatively with a favorable outcome.

### Case Report

A 32-year-old male patient presented in emergency department with the history of ingestion of two celphos tablets 7 hours ago. On examination, blood pressure was 76/54 mm of Hg; pulse rate was 114/minute and respiratory rate was 24/minute. SaO2 was 86% in ambient air which improved after oxygenation at the rate of 4-5 L/minute. GCS was 15/15 and without any focal neurological signs. Review of other systems was normal. Investigations were within normal limits except there was lactic acidosis on his arterial blood gas analysis. Patient was shifted to intensive care unit for management which included central venous pressure guided intravenous fluids, ionotropes and magnesium sulfate injections. On day two of admission, he was complaining of shortness of breath and passing of cola coloured urine. On examination, he was found to be pale. Icterus was present. All the investigations were repeated which showed a fall in the hemoglobin level from the initial baseline level with unconjugated hyperbilirubinemia. In view of unconjugated hyperbilirubinemia and rapid fall in hemoglibin level diagnosis of hemolysis was considered. History was reviewed for hemolytic anemia and it found unremarkable. He denied ingestion of any other drug. Patient was worked up for hemolytic anemia. Peripheral blood film revealed macrocytosis and anisopoikilocytosis. No schistocytes or red blood cells fragments were found. Reticulocyte production index was 2.9. G6PD activity levels were normal. Serum lactate dehydrgenase was 1900 U/L (normal: 250U/L), haptoglobin was 11mg / dL (normal 30-200mg/ dL). Direct Coomb's test was negative. Partial thromboplastin time and prothrombin time were normal. Urine showed presence of hemosiderin. Urine microscopy was normal. Ultrasonography abdomen was normal. Serial hemogram and biochemistry during hospitalisation and follow-up are shown in Table 1. During the hospitalisation no drugs were administered which may cause hemolysis. The patient remained afebrile. Patient took a discharge on request on fifth day of admission and reported back on day 10 of ingestion for follow up. He was refered for psychiatry evaluation.

## DISCUSSION

Phosphine (PH3) gas is the active ingredient of ALP. After ingestion, PH3 is liberated in contact with gastric acidic fluids, which is absorbed through the gastric mucosa. PH 3 inhibits mitochondrial oxidative phosphorylation due to inhibition of cytochrome C oxidase. It also causes reactive oxygen species over production, intracellular lipid peroxidation, vascular wall disintegrity, inhibition of cholinesterase activity, hemolysis, and methemoglobinemia thus leading into multiorgan failure ^2^. Hemolysis is a rare clinical presentation of aluminum phosphide poisoning. The reason for intravascualr hemolysis in patients with normal G6PD is conjectural at the moment. Hemolysis can be due to direct toxic effect or in the environment of metabolic acidoisis ^4,5^. Morphological changes in RBC have been reported in animal models. They are attributed to lipid peroxidation of RBC cell membrane ^6^. However, presence of normal RBC morphology on peripheral smear in our case counters this hypothsis. Similar observation was reported by Aggarwal et al.^5^ Hemolysis due to metabolic acidosis is also a possibilty. Despite the fact that metabolic acidosis is a common feature of ALP posoining intravascular hemolysis is not frequently reported. The argument in favour of metabolic acidosis could be that patients succumb to the poisoning before hemolysis is clinically evident. Another point is that whether hemolysis itself contributes to metabolic acidosis. Fresh RBC transfusion has ameliorated metabolic acidosis and enhanced survival in AlP-poisoned rats^7^. Hemoglobin has high buffering capacity and erythrocytes can potentially be used for management of metabolic acidosis.

**Table 1:** Laboratory data during hospital stay and follow-up

*Parameter*	*Day 1*	*Day 2*	*Day 3*	*Day 4*	*Day 10*
Hemoglobin (g%)	13.5	7.9	8.5	8.9	10.4
TLC (cells/mm^3^)	3890	5800	5900	5940	5330
Platelet (/mm^3^)	396000	226500	327000	431000	554700
Total bilirubin (mg/dL)	1.6	8.2	7.6	5.8	4.6
Unconjugated bilirubin (mg/dL)	0.8	7.9	7.3	5.5	4.3
AST (IU)	42	176	156	128	67
ALT (IU)	34	45	39	32	34
BUN (mg/dL)	12	32	28	21	13
Creatinine BUN (mg/dL)	0.7	1.9	1.7	1.3	1.2

*TLC:* Total leukocyte count, *AST:* Aspartate transaminase, *ALT:* Alanine transaminase, *BUN:* blood urea nitrogen

Aluminum poisoning can be complicated with intravascular hemolysis and should be considered in emegency department.
